# Underestimated Survival of *Campylobacter* in Raw Milk Highlighted by Viability Real-Time PCR and Growth Recovery

**DOI:** 10.3389/fmicb.2020.01107

**Published:** 2020-06-17

**Authors:** Imke F. Wulsten, Alibek Galeev, Kerstin Stingl

**Affiliations:** ^1^ National Reference Laboratory for Campylobacter, Department of Biological Safety, German Federal Institute for Risk Assessment (BfR), Berlin, Germany; ^2^ Institute of Medical Microbiology and Hospital Epidemiology, Hannover Medical School (MHH), Hannover, Germany

**Keywords:** propidium monoazide, viable but non-culturable, resuscitation, food safety, oxidative stress, real-time polymerase chain reaction

## Abstract

Raw milk is a frequent vehicle for transmission of thermophilic *Campylobacter*, leading to reported outbreaks. Milk is a challenging food matrix for pathogen detection, due to its high protein and lipid content. Limited detection of *Campylobacter* colony-forming unit (CFU) in raw milk might underestimate the pathogen’s infectious potential. We optimized a viability real-time PCR (qPCR) for application with raw milk. The procedure was robust against variations of milk lots and different *Campylobacter* strains. Various DNA-intercalating dyes were evaluated for their ability to reduce the PCR signal of dead cells. Only propidium monoazide (PMA) and PMAxx qualified for diagnostic use. Different sedimentation properties of viable and dead *Campylobacter jejuni* and *Campylobacter coli* strains in 10-fold diluted milk enhanced viable/dead differentiation. The new method enabled to review survival of *Campylobacter* spp. in raw milk based on viable cells harboring an intact cell membrane. The data were compared to culturability according to ISO10272-2:2017. A difference of up to 4.5 log_10_ between viable *Campylobacter* counts and CFU values became apparent. Relevance of viability qPCR values was corroborated by full recovery of CFU under extremely reduced oxygen concentration in the presence of hydrogen. Recovery of CFU was limited, however, upon prolonged exposure in raw milk. The data confirm that *Campylobacter* survival in raw milk can be largely underestimated when relying on CFU data only. We conclude that raw milk led to oxidative stress-induced growth arrest in thermophilic *Campylobacter*, which was reversible by reduction of the oxygen partial pressure in a time-limited way.

## Introduction

In the European Union, campylobacteriosis is currently the most frequent bacterial food-borne disease, with 246,571 reported human cases in 2018 (EFSA, 2019). Acute symptoms include watery or bloody diarrhea, abdominal cramps, vomiting, and fever. Associated chronic complications involve reactive arthritis, inflammatory bowel disease, and neurological disorders such as Guillain-Barré syndrome (Keithlin et al., 2014). Rare fatal cases were reported in children less than 5 years of age and in elderly and immunocompromised patients (EFSA, 2019).

Transmission of *Campylobacter* mainly occurs *via* contaminated food, by direct contact with colonized animals and also by untreated water (Kaakoush et al., 2015; Mughini-Gras et al., 2016; Rosner et al., 2017). Undercooked poultry meat and an insufficient kitchen hygiene related to raw meat handling are classical causes for the majority of sporadic cases. However, *Campylobacter* outbreaks are frequently reported upon consumption of raw milk (EFSA, 2018). Due to the repeal of the European milk quotas in 2015, farmers intensified the local sale of raw milk *via* milk-filling stations. In turn, consumer behavior changed in favor of an increased consumption of raw milk, neglecting prior heating recommendations. In Germany, 26% of all reported food-associated outbreaks with “high evidence” were caused by *Campylobacter* in 2018 (BVL, 2019). Most of them were caused by consumption of raw milk.

Cultural detection of *Campylobacter* in raw milk is difficult due to rapid decline of colony-forming unit (CFU) (Barrell, 1981; Doyle and Roman, 1982; Humphrey, 1986). Though considered fastidious in growth, *Campylobacter* are supposed to resist various environmental stresses. The genus has been suggested to outlast unfavorable environmental conditions in the viable but non-culturable (VBNC) state, putatively able to resuscitate and maintain its infective potential (Rollins and Colwell, 1986; Federighi et al., 1998; Baffone et al., 2006).

Real-time PCR (qPCR) offers a highly sensitive culture-independent quantification method. It can be combined with a DNA intercalating agent, which enters dead cells, crosslinks to DNA, thereby impeding DNA amplification during PCR. This way, viable cells with an intact membrane can be differentiated from dead cells (Nocker et al., 2006; Krüger et al., 2014; Pacholewicz et al., 2019). It allows determining the highest possible residual risk present, while the minimal risk is indicated by CFU.

We established a culture-independent procedure that allows quantification of viable thermophilic *Campylobacter* in raw milk employing “viability qPCR.” Applying this method, we investigated *Campylobacter*’s survival in raw milk with intriguing results; a substantial survival of *Campylobacter* was observed by viability qPCR, while CFU dropped to non-detectable levels. To support our findings based on viability qPCR, recovery of CFU was demonstrated by lowering the partial pressure of oxygen beyond ISO standard recommendations (ISO 10272-2:2017).

## Materials and Methods

### Strains and Growth Conditions

*Campylobacter jejuni* DSM 4688, *Campylobacter coli* DSM 4689, and *Campylobacter sputorum* DSM 5363 were derived from the DSMZ strain collection (DSMZ, Braunschweig, Germany). *C. jejuni* BfR-CA-13255, BfR-CA-13290, BfR-CA-14727, BfR-CA-15123, and BfR-CA-15146 had been isolated from raw milk by the Federal State Laboratories in Germany.

Strains stored at −80°C (MAST Group Ltd., Bootle, UK) were cultured on Columbia agar supplemented with 5% sheep blood (ColBA, Oxoid, Thermo Fisher Scientific Inc., Waltham, Massachusetts, USA) at 42°C under microaerobic conditions (5% O_2_, 10% CO_2_, and 85% N_2_) in a microaerobic incubator (Binder GmbH, Tuttlingen, Germany) for 24 h. After subculture for 18 ± 2 h, cells were suspended in Brucella broth (BB, Beckon Dickinsen, Franklin Lakes, New Jersey, USA) at OD_600_ = 0.2, corresponding to ~9 log_10_ cell counts per ml (Krüger et al., 2014). The cells were 10-fold diluted to reach 8 log_10_ cell counts per ml and kept on ice until further use (viable cells). The non-thermophilic *C. sputorum* was grown at 37°C on ColBA for initially 48 h starting from a −80°C cryoculture. It was subcultured for 24 h and used for the production of a dead cell standard according to Pacholewicz et al. (2019).

Quantification by CFU was performed according to ISO 10272-2:2017 with BB as diluent and plating was performed in duplicate on mCCDA (Oxoid, Thermo Fisher Scientific., Waltham, Massachusetts, USA). The theoretical sensitivity was 1 CFU/ml milk. The standard incubation occurred for 48–72 h at 42°C using an atmosphere of 5% O_2_, 10% CO_2_, and 85% N_2_ (a). Variation of atmospheric conditions was performed using incubators and gas replacement jars (Oxoid™ Anaerobia System, Thermo Fisher Scientific Inc., Waltham, Massachusetts, USA) filled with gas mixtures (Air Liquide, Paris, France). The detection limit for condition b–e. was 10 CFU/ml milk.

**Figure 5:**
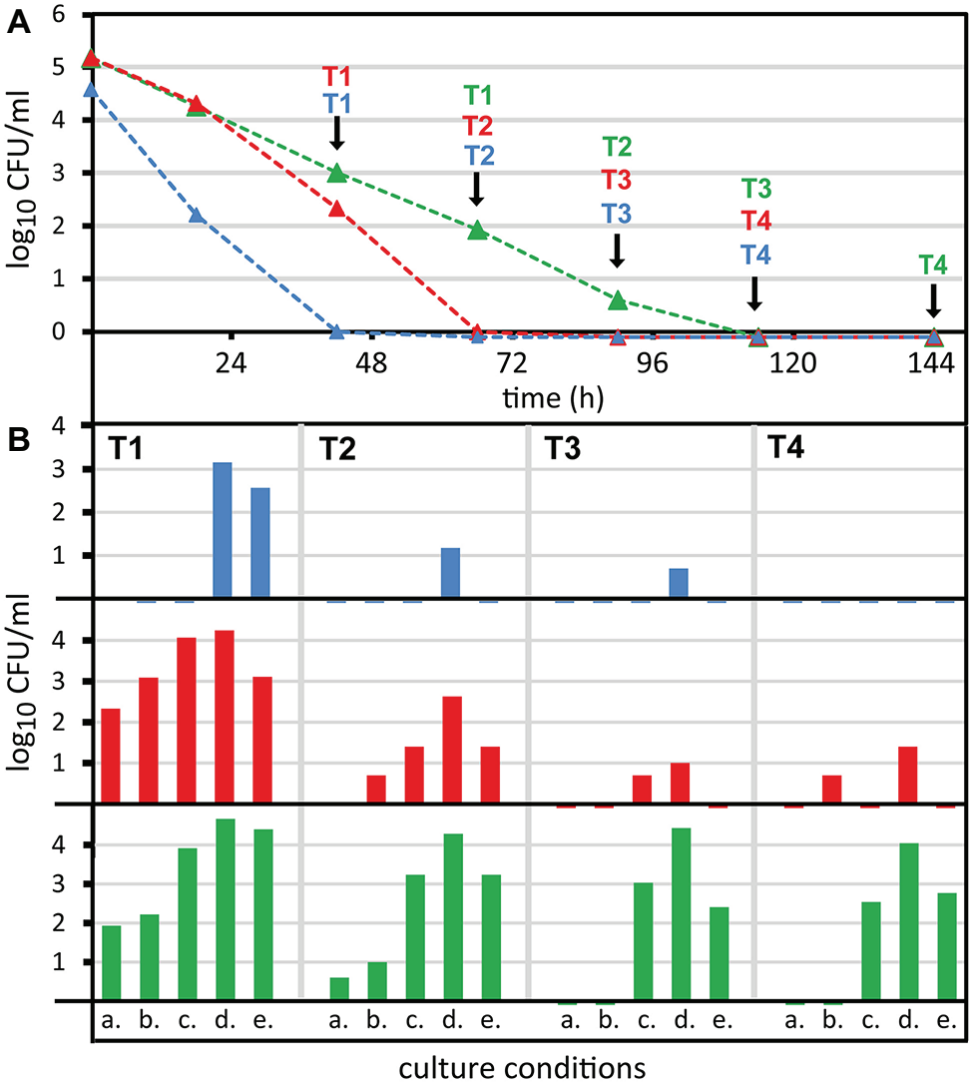
Recovery of non-culturable *Campylobacter* from raw milk by reduction of oxygen partial pressure. *Campylobacter* were spiked at 6 log_10_/ml bacterial counts into raw milk and stored refrigerated. **(A)** Colony-forming unit (CFU) was determined under standard conditions (5% O_2_, 10% CO_2_, rest N_2_ at 42°C, condition a.). **(B)** At selected time points (T1–T4) enumeration was performed under four alternative microaerobic culture conditions. Alternative culture conditions at 37°C and with N_2_ as background gas: b. 5% O_2_, 10% CO_2_; c. 1% O_2_, 10% CO_2_; d. 3.5% H_2_, 1% O_2_, 10% CO_2_; e. 3.5% H_2_, 6% O_2_, 7% CO_2_. Mean values of one representative experiment are shown for three strains (blue, *Campylobacter coli* DSM 4689; red, *Campylobacter jejuni* DSM 4688; green, *C. jejuni* BfR-CA-13290). Viability qPCR initially detected 5.0–5.6 log_10_/ml viable counts and 4.4–4.72 log_10_/ml after 144 h of exposure in raw milk for the three strains.

### Milk Lots and Spiking of the Samples

To assess the effect of milk batch variations, different batches of raw tank milk were either received from the institute’s own farming facility or collected from a regional milk producer. The fresh milk was kept at 4°C until processing, but not longer than 24 h after milking (*n* = 5). Frozen batches were stored at −20°C for less than 10 days (*n* = 4) or more than 450 days (*n* = 2). For all other experiments, one of the long-term frozen milk batches was used in order to improve comparability of the results.

Milk samples were spiked with *Campylobacter* strains to reach an initial concentration of 6 log_10_ cells/ml raw milk. The raw milk samples were kept in closed 50-ml tubes and incubated at 5 ± 1°C under normal atmospheric conditions. pH was stable and measured to be 6.7 ± 0.1 throughout the experiment. During a period of 6 days, samples were daily analyzed by CFU determination and viability qPCR analysis in parallel.

### Production of Dead and Transiently Inactive Viable Cells

If not otherwise stated, *C. jejuni* dead cell standards served as sample process control (SPC) during each experiment. Cells were killed by incubating 8 log_10_ cells in 100 μl PBS supplemented with 5% hydrogen peroxide (Carl Roth GmbH, Karlsruhe, Germany). After 1 h at room temperature, the cell suspension was diluted with 900 μl PBS and the sample was then centrifuged at 16,000 × *g* for 5 min. The cell pellet was resuspended in 1 ml PBS to obtain 8 log_10_ inactivated cells per ml, which were kept on ice until further use. In order to verify loss of CFU, 100 μl of each dead cell standard was plated on ColbA.

For the production of transiently inactive cells, the protonophor cyanide-m-chlorophenylhydrazone (CCCP, Sigma-Aldrich, Steinheim, Germany) was applied. An amount of 8 log_10_ cells in 1 ml PBS was incubated with 100 μM CCCP for 1 h on ice. We stained 6 log_10_ CCCP-treated cells with intercalating dyes, while keeping 10 μM CCCP during incubation. For CFU determination after recovery from CCCP stress, CCCP-treated cells were centrifuged at 16,000 × *g* for 5 min, once washed with 1 ml BB and plated as described above.

### Differentiation Between Viable and Dead Cells

From each raw milk sample, we took twice 1 ml; one of these two aliquots was processed without intercalating dye (total amount of cells) and the other was used with intercalating dye (only viable cells). Both aliquots were 10-fold diluted in 9 ml of precooled BB and centrifuged at 8,000 × *g* for 20 min at 4°C using 15 ml centrifugation tubes. The supernatant (BB with the major part of the milk components) was decanted and the remaining supernatant carefully withdrawn with a 1 ml slim-shaft micropipette. The pellet was immediately suspended in 1 ml PBS, transferred to a 1.5-ml tube, and stored on ice until staining with the DNA intercalating dye.

During all experiments, propidium monoazide (PMA, Biotium Inc., Hayward, California, USA) was applied as DNA intercalating agent. When indicated, ethidium monoazide (EMA, Biotium Inc., Hayward, California, USA), PMAxx (Biotium Inc., Hayward, California, USA), or PEMAX (GenIUL, Terrassa, Spain) were used. Intercalating agents were applied at a final concentration of 50 μM. Stock solutions were stored in 20% dimethyl sulfoxide at −20°C. In order to guarantee an efficient reduction of the dead cell signal, control samples either contained dead *C. jejuni* cells (SPC) or dead *C. sputorum* cells (ISPC) at 6 log_10_ cells/ml. The protocol was as previously described (Pacholewicz et al., 2019). In brief, samples were pre-incubated for 10 min at 30°C and 700 rpm in a thermomixer, before addition of the DNA intercalating agent, shortly vortexed, and further incubated for 15 min at 30°C at 700 rpm in the dark. Subsequently, samples were cross-linked for 15 min using the PhAST Blue photo-activation system (GenIUL, Terrassa, Spain) at 100% light intensity. Samples were centrifuged for 5 min at 16,000 × *g*, the supernatant was discarded, and the cell pellets were stored at −20°C until DNA extraction.

### DNA Extraction and qPCR

Genomic DNA was extracted using the GeneJet Genomic DNA Purification kit (Thermo Fisher Scientific Inc., Waltham, Massachusetts, USA) according to the manufacturer’s instructions and extracted DNA was eluted in 100 μl. qPCR was carried out on the same day using 10 μl of extracted DNA per reaction.

qPCR was performed targeting a fragment of the 16S rRNA gene of thermophilic *Campylobacter* or of *C. sputorum* (Pacholewicz et al., 2019). In short, the qPCR mastermix contained final concentrations of 1× Platinum Taq buffer, 2.5 mM MgCl_2_, 0.2 mM of each dNTP, 0.06 × ROX, 500 nM of each Jos-F1 and Jos-R1 primer, 300 nM of each IPC-ntb2-F and IPC-ntb2-R primer (HPLC-grade, Sigma-Aldrich, Steinheim, Germany), 100 nM of each dark-quenched probe (IPC-ntb2-P-TAMRA and Jos-P-FAM, TIB MOLBIOL, Berlin, Germany), 2U Platinum Taq DNA polymerase (Invitrogen, Thermo Fisher Scientific Inc., Waltham, Massachusetts, USA), and 25 copies of the IPC-ntb2 plasmid (Anderson et al., 2011) in a final qPCR reaction volume of 25 μl. qPCR was performed on a 7500 Real-Time PCR system (Applied Biosystems, Thermo Fisher Scientific, Waltham, Massachusetts, USA) using the 7500 Fast Software v2.3. The program started with 3 min at 95°C followed by 45 cycles of 15 s at 95°C, 1 min at 60°C and 30 s at 72°C. For the quantitative detection of *C. sputorum* the mastermix was as above but contained instead of Jos-F1 and Jos-R1 the primer pair Csput-F and Csput-R. Likewise, the Jos-P probe was exchanged with the *C. sputorum*-specific probe Csput-P-Cy5 as detailed in Pacholewicz et al. (2019). The sequences of the primers and probes (quenched by the black berry quencher, BBQ) were as follows: Jos-F1 (5′-CCTGCTTAACACAAGTTGAGTAGG-3′), Jos-R1 (5′-TTCCTTAGGTACCGTCAGAATTC-3′), Jos-P (6FAM-TGTCATCCTCCACGCGGCGTTGCTGC-BBQ), IPC-ntb2-F (5′-ACCACAATGCCAGAGTGACAAC-3′), IPC-ntb2-R (5′-TACCTGGTCTCCAGCTTTCAGTT-3′), IPC-ntb2-P (TAMRA-CACGCGCATGAAGTTAGGGGACCA-BBQ), Csput-F (5′-TGGGAAATGTAGCTCTTAATAATATATATC-3′), Csput-R (5′-CCTTACCAACTAGCTGATACAATATAG-3′), and Csput-P (Cy5-CCTCATCCCATAGCGAAAGCTCTT-BBQ).

Each qPCR run contained genomic standards, comprising five decile serial dilutions ranging from 50,000 to 5 genomic copies per reaction in duplicate. The genomic standard was freshly reconstituted from dried stabilized DNA aliquots as described previously (Pacholewicz et al., 2019).

### Statistical Analysis

Data were analyzed using GraphPad Prism v. 5.01 software. Statistical analysis was performed using the Mann-Whitney *U*-test. Graphs display the mean values ± SD, unless stated otherwise.

## Results

### Establishing Quantification of Viable *Campylobacter* in Raw Milk by qPCR

We intended to answer the crucial food safety question whether the recovery of thermophilic *Campylobacter* from raw milk samples by CFU underestimates viable *Campylobacter* cells. We previously improved a culture-independent viability qPCR optimized for chicken rinses (Pacholewicz et al., 2019). As expected, direct quantification from undiluted raw milk samples was unsuitable because of two reasons – the bacteria could not be centrifuged in a quantitative manner probably due to high protein and lipid contents, and the opaque nature of raw milk rendered staining and proper crosslinking of DNA in dead bacteria inefficient.

**Figure 1:**
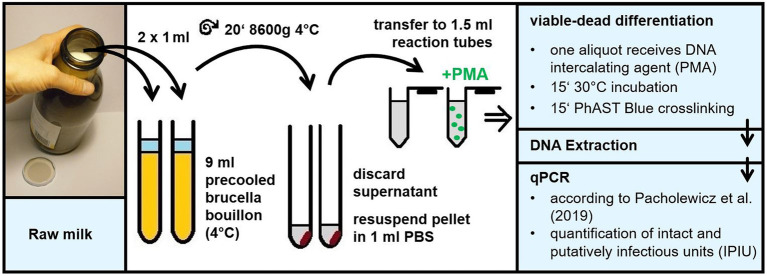
Schematic overview of the culture-independent method for quantification of viable *Campylobacter* in raw milk. The method comprises the following steps. Raw milk is diluted 10-fold in precooled Brucella broth to enable quantitative bacterial centrifugation. After careful withdrawal of non-pelleted raw milk residues, the pellet is resuspended in 1 ml PBS for viable/dead differentiation according to Krüger et al. (2014). Subsequently, the DNA intercalating agent is applied to block DNA of dead bacteria from PCR amplification. After DNA extraction qPCR is performed targeting the 16S rRNA sequence in intact and putatively infectious units of thermophilic *Campylobacter* according to Pacholewicz et al. (2019).

**Figure 2:**
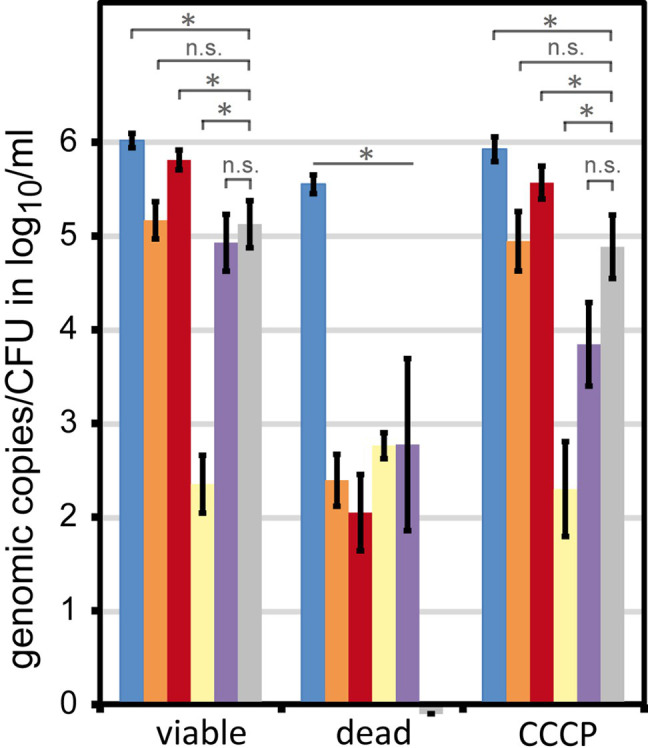
Signal reduction in dead and transiently inactive *Campylobacter* by different DNA intercalating agents. *C. jejuni* DSM 4688 were grown on ColbA for 18–20 h (viable bacteria). The bacteria were either killed by H_2_O_2_ or transiently inactivated using the protonophore CCCP. Fifty µM of the respective DNA intercalating agent was applied to 6 log_10_ bacteria per ml PBS. Columns display mean values ± standard deviation of genomic copies (blue, without treatment; orange, propidium monoazide (PMA); red, PMAxx; yellow, EMA; purple, PEMAX) or CFU (gray bars); dataset includes five independent experiments; ^*^ *p* < 0.01; n.s., non-significant (two-tailed Mann-Whitney *U*-test).

Thus, we diluted raw milk samples 10-fold in precooled BB in order to quantitatively harvest *Campylobacter* upon centrifugation and to reduce the amount of milk compounds putatively deteriorating the staining procedure (Figure 1). After resuspension of the bacterial pellet, various DNA intercalating dyes were tested. A proper method would lead to efficient elimination of the DNA amplification signal of dead cells. Meanwhile, it would most effectively amplify DNA of viable cells, even though these viable cells were transiently inactive in terms of growth. We wondered if the recently commercialized PMAxx and PEMAX agents had improved properties in the viable/dead differentiation protocol. In comparison with the known agents PMA and EMA, we examined their performance in the viability qPCR (Figure 2). For this purpose, *C. jejuni* DSM 4688 grown on ColBA for 18–20 h was resuspended in BB and used as viable untreated cells. Dead bacteria were produced by inactivation *via* oxidative stress as previously described (Krüger et al., 2014). Transiently inactivated cells were produced by treatment with 100 μM of the protonophor CCCP, abolishing the proton-motif force of the bacterium. Subsequently, 6 log_10_ per ml of these differently treated cells were stained with the various dyes and processed *via* qPCR and CFU determination (Figure 2).

Viable cells showed nearly identical CFU and live qPCR counts (5.17 ± 0.2 log_10_ live bacteria per ml and 5.13 ± 0.25 log_10_ CFU per ml qPCR counts in the presence of PMA; Figure 2, orange and gray bar). Around 10-fold more total genomic copies were detected *via* qPCR (6.02 ± 0.08 log_10_ per ml without any DNA intercalating dye; Figure 2, blue bar). These results are in agreement with previous data, demonstrating that only around every tenth *C. jejuni* DSM 4688 precultured under similar conditions displayed CFU capability (Krüger et al., 2014). CCCP-treated cells nearly fully restored their ability to form CFU upon release of CCCP stress (4.89 ± 0.34 log_10_ CFU/ml; Figure 2, gray bar) and qPCR results with PMA very well-matched CFU determinations (4.94 ± 0.32 log_10_/ml). In contrast, cells inactivated with 5% H_2_O_2_ (dead) were not able to form any CFU (Figure 2, gray bar). Likewise, all tested DNA intercalating agents reduced the detection of dead cells by ≥2.8 log_10_/ml genomic copies. This confirmed their ability to efficiently reduce the signal of dead cells under the tested conditions.

As observed before (Krüger et al., 2014) and also suggested for other bacteria (Nocker et al., 2006; Codony et al., 2015), EMA was indicative of metabolic activity and was not passively excluded from viable cells. Consistently, EMA largely underestimated transiently inactivated *C. jejuni* (2.35 ± 0.31 log_10_/ml and 3.84 ± 0.44 log_10_/ml genomic copies, respectively) and apparently entered viable bacteria. PMAxx led to slightly higher qPCR results than displayed by CFU, while PEMAX led to lower quantification of transiently inactivated (CCCP-treated) bacteria.

These observations indicate that PMA and PMAxx were passively excluded from intact bacteria. In contrast, PEMAX partially entered de-energized cells, but with less efficiency than EMA. Hence, we confirmed that PMA was the most suitable agent for viable/dead differentiation of *C. jejuni* cells.

### Variation of Milk Lots and *Campylobacter* Strains

**Figure 3:**
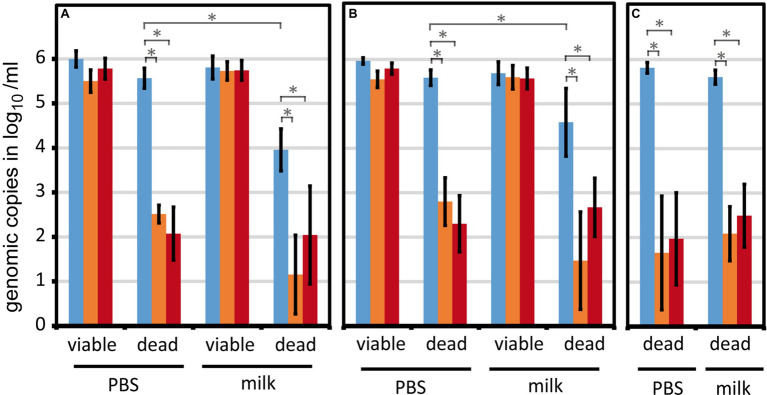
The viability qPCR method is robust against different milk lots and strains variations. Viable or H_2_O_2_-inactivated *Campylobacter* were spiked at 6 log_10_/ml into PBS or raw milk. Genomic counts were determined by qPCR upon differentiation of viable and dead cells using PMA or PMAxx. **(A)** Eleven different lots of raw milk (either fresh or after −20°C storage) were tested using the strain *C. jejuni* BfR-CA-13290; **(B)** Seven different *Campylobacter* strains (two reference strains and five field strains) were analyzed using one milk lot. **(C)** Five experiments were performed with H_2_O_2_-inactivated *C. sputorum* DSM 5363 as potential internal sample process control (Pacholewicz et al., 2019). Absence of rest signal from dead cells was defined as −0.1 log_10_/ml for enabling calculations. Blue, no treatment; orange, PMA; red, PMAxx. Columns display mean values ± standard deviation. ^*^ *p* < 0.01; n.s., non-significant (two-tailed Mann-Whitney *U*-test).

We scrutinized the viable/dead differentiating qPCR with regard to reproducible results upon matrix and strain variations. For this reason, different milk lots (*n* = 11; Figure 3A) and different *Campylobacter* strains (*n* = 7, including six *C. jejuni* and one *C. coli*; Figure 3B) were included in the experiments.

Viable cells of *C. jejuni* BfR-CA-13290 were reliably quantified in both PBS and raw milk using PMA or PMAxx, irrespective of various milk lots (Figure 3A). Likewise, different thermophilic *Campylobacter* strains could also be consistently quantified with only minor variations (Figure 3B). Interestingly, we observed a partial loss (~2 log_10_) of dead *C. jejuni* by centrifugation, which was strain-dependent (Figures 3A,B, dead, blue bars) and most pronounced for strain BfR-CA-13290. In contrast, viable cells were reproducibly retrieved independent of the strain tested. Hence, in combination with PMA or PMAxx treatment, viable/dead differentiation was even improved in raw milk compared to PBS. Dead *C. sputorum* cells, previously established as internal sample process control (ISPC) (Pacholewicz et al., 2019), were quantitatively retrieved from milk samples (Figure 3C), rendering this ISPC suitable for monitoring DNA loss for the reduction of the dead cell signal in raw milk.

### Survival of *Campylobacter* in Raw Milk at Refrigeration Temperatures

**Figure 4:**
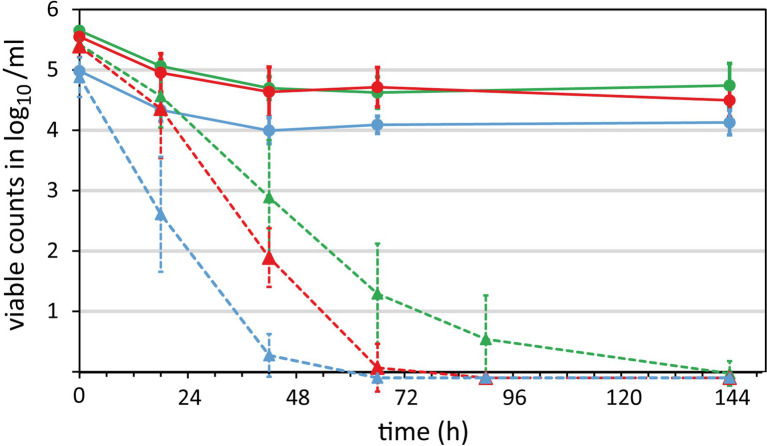
Survival of *Campylobacter* in raw milk. Three *Campylobacter* strains were spiked at 6 log_10_/ml bacterial counts in raw milk and stored aerobically at refrigeration temperatures. Samples were taken over time to quantify the survival by viability qPCR (solid lines) and CFU according to ISO 10272-2:2017 (dotted lines). Blue, *C. coli* DSM 4689; red, *C. jejuni* DSM 4688; green, *C. jejuni* BfR-CA-13290. Absence of CFU or genomic copies was defined as −0.1 log_10_/ml for enabling calculations. Mean values ± standard deviations are shown from at least five independent experiments.

With the new method at hand, we studied the survival of three *Campylobacter* strains in raw milk stored under aerobic conditions at 5 ± 1°C. Two reference strains (*C. jejuni* DSM 4688 and *C. coli* DSM 4689) and the outbreak-associated strain isolated from raw milk (*C. jejuni* BfR-CA-13290) were each spiked at an initial inoculum of 6 log_10_/ml. The milk samples were daily analyzed over a period of 6 days by viability qPCR and CFU determination (Figure 4).

As expected, CFU significantly decreased over time in a strain-dependent manner. CFU of *C. coli* DSM 4689 rapidly declined and the strain was uncultivatable within 3 days (Figure 4, dashed blue line). The CFU of *C. jejuni* BfR-CA-13290 declined most slowly and the strain was undetectable after 6 days in most experiments (Figure 4, dashed green line). CFU loss kinetics of *C. jejuni* DSM 4688 was intermediate of *C. coli* and the *C. jejuni* outbreak strain (Figure 4, dashed red line).

In contrast, the amount of intact and putatively infectious units (IPIU) only marginally decreased by around 1 log_10_/ml for the two *C. jejuni* strains and to a level of ~4 log_10_/ml for the *C. coli* strains during the first 2 days. Subsequently, IPIU remained on these levels throughout the whole experiment (Figure 4, solid lines). At the end of the experiment, we observed a large discrepancy between CFU and IPIU for all three strains, i.e., absence of CFU and still log 4–4.5 IPIU detected by viability qPCR.

### Recovery of CFU at Altered Culture Conditions

We questioned whether the observed prolonged survival in raw milk as assessed by viability qPCR reflected a real existent food safety issue, i.e., that bacteria were indeed viable. For this purpose, we intended to identify parameters critical for *Campylobacter* growth after exposure to raw milk.

We selected consecutive time points at which several growth conditions were tested in parallel. *Campylobacter* as a microaerobic organism is susceptible to oxidative stress outside its multiplication site. Thus, we tested whether altered temperature and atmospheric conditions can revert its capability to form CFU *in vitro*. We tested four alternative conditions (Figure 5B) in parallel to the standard condition of 42°C and an atmosphere of 5% O_2_, 10% CO_2_, and rest N_2_ (a). Condition b only differed by reduction of the temperature to 37°C. Furthermore, we additionally changed the concentrations of O_2_ (1% for c and d, 6% for e), CO_2_ (7% for e), and eventually added hydrogen (3.5% for d and e). The incubation time for optimal colony counting varied between the conditions. In the presence of 5–6% oxygen (a, b, and e), growth of *Campylobacter* led to well visible colonies after 2–3 days. It was confirmed that a prolonged incubation time did not lead to additional CFU. In contrast, at reduced oxygen levels (c and d), colonies grew slower, so that CFU were optimally counted after 4–5 days.

Alteration of atmospheric conditions significantly increased CFU in all strains tested in a time point‐ and strain‐dependent manner (Figure 5, conditions c–e). Reduced temperature eventually increased CFU recovery at early time points in *C. jejuni* strains, but the extent of this increase was less than 1 log_10_/ml (Figure 5, condition b). The condition, in which hydrogen was present and oxygen was reduced to 1%, resulted in maximal CFU detected for all strains (Figure 5, condition d). In comparison with the original condition (Figure 5, condition a) reduction of oxygen and addition of hydrogen maximally increased CFU by log 4.5 log_10_/ml in *C. jejuni* BfR-CA-13290 after 6 days of exposure in milk. Hence, the gap between the initial CFU read-out under standard conditions and the viability qPCR read-out of IPIU was completely bridged (Figure 4). In the other two strains, CFU increased at most by 2.5 (at T2, 66 h) and 3 log_10_ per ml (T1, 42 h) for *C. jejuni* DSM 4688 and *C. coli* DSM 4689, respectively. However, stimulating CFU recovery under reduced oxygen conditions in the presence of hydrogen was dependent on the time point of investigation, since the magnitude of CFU increase diminished with exposure time of *Campylobacter* in raw milk at 5 ± 1°C.

Our experiments demonstrated, for the first time, that the measured number of IPIU determined by viability qPCR are indeed relevant, since we found distinct conditions under which CFU could completely be restored.

## Discussion

Raw milk is an important vehicle for *Campylobacter* transmission and frequently involved in reported outbreaks. The raw milk matrix is challenging from the food safety perspective, since *Campylobacter* are difficult to be detected due to rapid decline in CFU. Current alternative methods target detection of *Campylobacter* after enrichment of raw milk in broth (Razzuoli et al., 2018) or by using in-line milk filters with higher pathogen contamination (FSAI, 2015; Artursson et al., 2018). To our knowledge, no culture-independent direct quantification method is so far available for raw milk. In the present study, we established a viability qPCR method, which allows quantifying IPIU of *Campylobacter* in raw milk. Furthermore, survival of *Campylobacter* spp. in raw milk was reassessed by qPCR and CFU. The proposed method was found to be robust against different milk batches and also suitable for different *C. jejuni* strains, the major species responsible for campylobacteriosis (EFSA, 2019).

While the standardized plate count approach has a quantitative sensitivity of 10 CFU/ml according to ISO 7218:2014, our qPCR-based method is 10-fold less sensitive due to limits of PMA staining and DNA extraction. However, we showed that qPCR can detect 4.5 log_10_/ml more viable *C. jejuni* in comparison to CFU enumeration under standard culture conditions (Figure 5). The results indicated an initial 1 log_10_/ml reduction in viable cells of the outbreak-associated *C. jejuni* strain within the first 42 h in raw milk. Notably, the remaining population appeared to cease their capacity to form colonies under normal growth conditions. Nevertheless, genomic DNA was protected by an intact cell membrane, which was inaccessible by PMA. In order to prove that qPCR results of viable bacteria are relevant for food safety, we bridged the gap between CFU and viability qPCR.

According to the standard protocols for enhanced enrichment of stressed *Campylobacter*, growth temperature is decreased for several hours to 37°C (ISO 10272-1:2017) based on observations of improved CFU recovery after cold stress (Humphrey, 1986). However, the effect of temperature reduction from 42°C to 37°C on increased *C. jejuni* CFU levels after raw milk exposure was negligible (Figure 5B, condition b).

Importantly, despite of decreasing CFU under standard conditions, an extremely low oxygen partial pressure (1% O_2_ in combination with 3.5% H_2_) reactivated *Campylobacter* to quantitatively grow on mCCDA (Figure 5B). These results contradict the traditional interpretation that reduced CFU necessarily reflect reduced viability of the pathogenic bacterium and demonstrate, for the first time, that “viability qPCR” detected IPIU indeed mirror viable cells.

Interestingly, addition of hydrogen (Figure 5, condition c) or lowering oxygen concentration (Figure 5, condition e) both stimulated growth of *Campylobacter*. The combination of hydrogen and low oxygen concentration had a cumulative effect on CFU recovery (Figure 5, condition d). Since hydrogen functions as electron donor of the respiratory chain (van der Stel et al., 2017), it may also enhance respiratory activity and further consumption of oxygen within the cell. Putatively, these results hint to the presence of a yet unknown oxidative stress response sensor, which regulates replication and/or division in thermophilic *Campylobacter*. It was shown previously that *C. jejuni* adapts to various microaerobic atmospheres by significantly changing transcription of specific genes, e.g., involved in detoxification (John et al., 2011). Furthermore, *C. jejuni* showed prolonged survival under atmospheric conditions in the presence of the oxygen-consuming bacterium *Pseudomonas* spp. (Hilbert et al., 2010).

Nevertheless, the potential to recover CFU by drastic decrease of oxygen was diminished with time of exposure in milk, suggesting further yet unknown signals/regulators for entry and exit of the non-culturable state of *Campylobacter*.

## Conclusion

The presented work illustrates the underestimation of *Campylobacter* survival by standard plate count conditions in raw milk and calls for complementary culture-independent quantification of viable cells to improve risk assessment. Incubation under lowered oxygen tension in the presence of hydrogen was demonstrated to improve *Campylobacter* detection from raw milk in a time-limited way. Consequently, risk assessment of contaminated foods based on CFU might lead to an underestimation of the actual risk to human health. Future studies will decipher further parameters for reactivating *Campylobacter* culturability upon stress exposure in food matrices and in the environment. They will reveal so far unknown transmission routes as well as persistence niches of the versatile food-borne pathogen.

## Data Availability Statement

All datasets generated for this study are included in the article.

## Author Contributions

IW performed experiments, analyzed data and co-wrote the manuscript. AG performed experiments. KS designed the study, analyzed data and co-wrote the manuscript.

## Conflict of Interest

The authors declare that the research was conducted in the absence of any commercial or financial relationships that could be construed as a potential conflict of interest.
